# Knee pain in young adult women- associations with muscle strength, body composition and physical activity

**DOI:** 10.1186/s12891-021-04517-w

**Published:** 2021-08-21

**Authors:** Ylva B Ericsson, Fiona E McGuigan, Kristina E Akesson

**Affiliations:** 1 Department of Clinical Sciences, Lund University, Malmö, Sweden; 2 Department of Ortopedics, Skane University Hospital, 205 02 Malmö, Sweden

**Keywords:** Knee pain, Knee osteoarthritis, Thigh muscle strength, Body composition, Young women

## Abstract

**Background:**

Knee pain is studied mostly in older age groups, although in young adults it may be an indicator of future impaired musculoskeletal health. Therefore, the aim of this study was to examine the longitudinal association between knee pain and thigh muscle strength in young adult women and to explore the associations between muscle strength, body composition, physical activity and knee pain.

**Methods:**

The PEAK-25 cohort consists of women aged 25 at baseline (N=1064). At the 10-year follow-up n=728 attended for DXA-measured body composition and muscle strength assessment and n=797 answered the questionnaire on health and lifestyle. Independent samples t-test was used to compare women with and without knee pain, Spearman correlation was used to test the longitudinal association between strength and knee pain.

**Results:**

Knee pain was reported by one third of the women at follow-up (n=260, 33%), although physical activity levels were similar in those with and without pain (high level 50 vs 45 % (*p*= 0.18). Body composition differed, however. Women with knee pain had higher BMI (25.6 vs 24.1), fat mass index (9.2 vs 8.2) and % total body fat mass (34.7 vs 33.2). Simultaneously, they had lower % lean mass (total body 61.5 vs 62.8; legs 20.6 vs 21.0) and lower thigh muscle strength (extensors 184.9 vs 196.8, flexors 96.6 vs 100.9, *p*<0.05), but slightly higher hamstrings-to -quadriceps ratio (0.53 vs 0.51, *p*=0.04). Muscle strength at baseline weakly correlated with knee pain at follow-up (extensor r_s_= -0.04; flexor -0.02, *p*>0.2). Overweight women had higher absolute thigh muscle strength, but lower weight-adjusted strength than normal weight women (*p*<0.001). Leg lean mass explained 26-34% of the variation in muscle strength and adjustment for physical activity level had little effect.

**Conclusion:**

Knee pain is already common among women in their mid-thirties. Lower thigh muscle strength in the mid-twenties was not associated with future knee pain, however women with knee pain tended to have lower thigh muscle strength and a body composition of higher body fat combined with lower lean mass. Maintaining a healthy body composition and adequate thigh muscle strength may be beneficial for knee joint health.

**Supplementary Information:**

The online version contains supplementary material available at 10.1186/s12891-021-04517-w.

## Background

Knee pain is common at all ages, but prevalence increases with age [1]. In a Swedish population-based study of individuals aged 56-84, 25 % reported frequent knee pain [2]. Women aged 50 years or older have a higher prevalence of knee pain than men, but in younger age groups both men and women have similar frequency of knee complaints [1]. The prevalence of knee pain and symptomatic knee osteoarthritis (OA) has, for reasons unknown, increased substantially during the last decades, while the prevalence of radiographic knee OA has not [3].

Knee pain may start with a knee injury, such as a ruptured ligament or a torn meniscus, but it may also arise without preceding trauma. In young women patellofemoral pain is common [4] and may increase the risk of femuropatellar osteoarthritis [5]. Known risk factors for knee pain are the same as for knee OA; heredity, female gender, higher age and knee injury, overweight and occupational high joint loading [6–9], this also includes muscle weakness [10, 11].

Muscle strength is crucial for physical function and strength levels differ between individuals according to age, sex and body composition. Heritable factors explain approximately 50% of an individual’s muscle strength, with the influence of environmental factors increasing with age, since physical activity and training habits during the life span affect muscle strength [12]. Knee extensor muscle weakness has been associated with worsening of knee pain in individuals with or at risk of developing knee OA [11]. It has also been associated with cartilage loss in individuals with knee pain [10]. In theory, adequate knee extensor and flexor muscle strength is important for the knee joint since these have a stabilizing and shock-absorbing function that may protect the cartilage from microtrauma and high peak loads during gait and physical activity [13, 14]. Furthermore, relative weakness of the knee flexors in comparison to the knee extensors have been associated with inferior muscular stability and increased risk of knee injury and knee OA [15]. Despite this, it is still not established whether strong muscles can protect against development of knee OA, as previous studies are contradictory [8, 16].

Muscle strength in the lower extremities is associated with body weight and body composition. Previous studies have shown that overweight women generally have higher muscle strength in the lower extremity than their normal weight counterparts, at least in anti-gravitational muscles such as the knee extensors; this is thought to be a training effect from the extra load during everyday activities [17]. However, when adjusting strength for body weight, which is the ordinary procedure in research, the relationship often is inversed [18]. Body composition consisting of low percentage lean mass and high percentage fat mass has been suggested to be a risk factor for knee OA [19, 20], especially in women [21, 22]. Lean mass is highly correlated with muscle strength, and with age, decreases more slowly than strength [23]. Interestingly, lower lean mass in the legs has been associated with knee pain in people with knee OA [24], and a decline in leg lean mass has been reported in women with early knee OA [25].

Knee complaints such as joint swelling, stiffness and pain during load-bearing activities affects physical function negatively and may interfere with leisure activities and activities of daily life. In the young and middle-aged knee pain, even if not severe enough to seek medical attention, could hinder participation in sport and physical exercise and if long-term may lead to adverse effects on general health. Most studies on non-traumatic knee pain have focused on older people. However, in younger women knee pain may be an early indication detrimental to future musculoskeletal health [26].

Based on this rationale, and with the hypothesis that strong thigh muscles (i.e. knee extensors and knee flexors) may protect against knee pain we examined the longitudinal association between thigh muscle strength and knee pain in a population-based cohort of young adult women, assessed at age 25 and again 10 years later. We also explored, cross-sectionally, association between muscle strength, body composition, physical activity and knee pain. To our knowledge, this is the only study to investigate the prevalence of knee pain and associated muscle strength and body composition in women in their mid-twenties to mid-thirties.

The aims of the study were to: (i) compare women with and without knee pain at follow-up in relation to thigh muscle strength, body weight, body composition and self-reported physical activity level; (ii) examine the association between muscle strength in women at age 25 and knee pain at follow-up and;(iii) explore associations between muscle strength, body composition and physical activity level at baseline

## Methods

The PEAK-25 cohort is a longitudinal population based cohort of young adult women [27]. The main purpose of the cohort is to study the relationship between genetic, clinical and lifestyle factors associated with attaining peak bone mass. The sub-study described here focuses on knee joint pain, muscle strength and body composition. Briefly, 25-year old women living in Malmö, Sweden, were invited to the study by letter during 1999-2004. Criteria for inclusion in the study was being exactly 25 years of age. The sole exclusion criteria applied was pregnancy, either currently or during the 12 months prior to inclusion. At baseline, 1166 of 2394 agreed to participate, 102 individuals were excluded due to pregnancy, leaving a cohort of 1064 women (mean age 25.00).

These participants were subsequently invited to a 10-year follow-up investigation; 728 (68%) attended the investigation, while 797 (75%) completed the questionnaire. Women unable to participate due to pregnancy or breast-feeding, were invited to attend at a later date. Participants were extensively investigated at each visit and questionnaires provided detailed information on lifestyle and health.

The study was approved by the Ethical review board at Lund University and performed in compliance with the Helsinki declaration. All participants gave written informed consent.

### Knee pain

Physical examination was not part of the study protocol, therefore at follow-up, we relied on capturing the ‘personal perception’ through a self-administered questionnaire to assess knee pain and other knee complaints/symptoms (e. g. swelling, grinding/clicking noises from the knee joint, stiffness) during the previous month. The questionnaire, based on the American college of Rheumatology (ACR) criteria for clinical diagnosis of knee OA [28] consisted of seven questions with five response alternatives each, as shown in Additional file 1.

### Muscle strength

Isokinetic muscle strength in the knee extensors and flexors of the right leg was measured using a computerized dynamometer, Biodex System 3 [29]. Muscle strength was measured as concentric peak torque at the velocity of 60°/s in the range 10°-90° knee flexion. Participants were instructed to make a maximal effort, i.e. to push as hard as they could both in extension and in flexion, and verbal encouragement was given throughout. Participants completed one repetition with concentric extension and flexion to become familiar with the test. After the practice, five repetitions where completed. Peak torque (the highest value of the best repetition) was recorded, and results for knee extensors and knee flexors was reported both as absolute strength in Newton meter (Nm) and adjusted for body weight (%) according to the formula: peak torque (Nm)/weight (kg) x 100. The hamstrings-to-quadriceps strength ratio was calculated according to the formula: knee flexor strength (Nm)/ knee extensor strength (Nm).

### Body composition

Total body composition was measured by Dual Energy X-ray Absorptiometry (DXA) (Lunar Prodigy, GE Healthcare Lunar, Madison, Wisconsin, USA). In this study we related lean mass (kg) and fat mass (kg) to total body mass and used percent lean mass body (% lean mass-total body), percent lean mass legs (% lean mass-legs), and percent fat mass body (% fat mass-total-body) in the analyses. In addition to BMI (kg/m^2^), a fat mass index (FMI) was calculated (total body fat mass (kg)/height (m)^2^) since this is believed to be a more accurate measure of overweight [30].

### Self-reported physical activity

In the questionnaire, participants selected one of four alternatives to grade their general level of physical activity during the previous month. Grade 1) Not physically active (besides short walks and light gardening); Grade 2) Low intensity physical activity a couple of hours/week (long walks, normal gardening, bicycling, dance etc); Grade 3) Moderate intensity physical activity a couple of hours/week (tennis, swimming, running, gym training, football etc); Grade 4) Vigorous physical activity that requires a considerable effort most days a week. Based on these, self-reported physical activity was categorized as low (grades 1 and 2) or high (grades 3 and 4).

### Statistical analysis

As our primary aim was to assess the relationship between knee pain at follow-up and other factors (muscle strength, body composition and physical activity) we defined knee pain as “having knee symptoms at least monthly”. To compare characteristics in women with and without knee pain we used Independent samples t-test for continuous variables (weight, BMI, FMI, percentage of lean and fat mass) and Pearson’s Chi Square test for categorical data (physical activity level, knee injury, parent with knee OA) (Table 4).

To investigate the association between body composition, knee extensor and flexor strength at baseline we used the FMI classification ranges for women by Kelly et al [31] to categorize participants as normal weight (FMI ≤ 9 kg/m^2^), overweight (FMI >9 – 13 kg/m^2^ ) or obese (FMI >13 kg/m^2^). One-way ANOVA and Tukey’s post hoc test was used to compare the categories (Table 2).

Spearman correlation analysis was performed to test the association between knee extensor/knee flexor strength at baseline and frequency of knee pain at follow-up as well as to test the association between muscle strength, leg lean mass and physical activity.

Linear regression analyses were performed to test the association between knee extensor/knee flexor strength and leg lean mass, adjusting for physical activity level (Table 3). To avoid multi-collinearity knee extensor strength and knee flexor strength were analyzed separately.

To review the possibility of selection bias between those who attended follow-up and those who dropped out, we compared their baseline characteristics using Independent samples t-test, Mann Whitney U test and Pearson’s Chi Square. The N=270 lost to follow-up had, a higher mean body weight and BMI but did not differ in terms of muscle strength, physical performance or physical activity level.

A *p*-value of <0.05 was considered nominally significant. All statistical analyses were performed using IBM SPSS Statistics for Windows, version 24.0 (IBM Corp., Armonk, N.Y., USA).

## Results

### General characteristics at baseline and follow-up

The characteristics of those attending baseline and those attending both initial and follow-up visits are presented in Table 1.

**Table 1:** Characteristics of study participants at baseline and follow-up

	Baseline *n*=1064		10-yr follow-up *n*=728–797^a^	
*Age (yrs)*	25.5	(0.2)	36.5	(0.8)
***Knee muscle strength***				
Extensors, absolute (Nm)	141.8	(27)	131.6	(26.5)
Extensors, weight-adjusted (%)	221.9	(36.7)	192.8	(36.6)
Flexors, absolute (Nm)	71.9	(15.6)	67.8	(16)
Flexors, weight-adjusted (%)	112.6	(22)	99.3	(22.4)
Hamstrings-to-quadriceps ratio	0.51	(0.08)	0.52	(0.09)
***Body composition***				
Weight (kg)	64.7	(11.4)	69.3	(13.2)
BMI ((kg/m^2^)	23	(3.8)	24.6	(4.5)
FMI (total body fat mass (kg)/height (m)^2^	7.6	(3)	8.5	(3.4)
Fat Mass-total body (%)	31.5	(0.07)	33.7	(0.07)
Lean Mass-total body (%)	63.3	(0.07)	62.4	(0.07)
Lean Mass-legs (%)	21	(0.03)	20.9	(0.02)
High physical activity level, n (%)	826	(78)	371	(46)
Low physical activity level, n (%)	228	(22)	427	(54)
Knee injury, n (%)	342	(32)	120	(15)

Data is presented as mean (SD) unless otherwise indicated

^a^728 participants attended for Biodex/DXA; 797 returned questionnaires (knee health/physical activity)

According to BMI-classification the proportion being overweight and obese increased between baseline and follow-up; 17% overweight vs 23% and 5% obese vs 12%. This closely mirrors the FMI-classification of 18% vs 23% overweight and 6% vs 11% obese. Muscle strength declined over the 10-years. Knee extensor absolute strength was 7% lower (131.6 vs 141.6 Nm) and 13% lower after adjustment for body weight. Corresponding figures for knee flexor strength were 6% (67.8 vs 71.9) and 12% lower, respectively. The hamstrings- to quadriceps strength ratio was similar at baseline and follow-up, mean (SD) 0.51 (0.08) and 0.52 (0.09), respectively. Physical activity changed over time; No activity (Grade 1) 2% baseline vs 12% follow-up, light activity (grade 2) 18% vs 41%, regular exercise (Grade 3) 72% vs 40% and strenuous exercise (Grade 4) 8% vs 7%.

A history of knee injury was reported by almost one third at age 25 (342/1064). At the 10-year follow-up it was lower, at 15% (120/797), whereof 7.8% reported having undergone knee surgery.

### Association between muscle strength and body composition

**Fig. 1:**
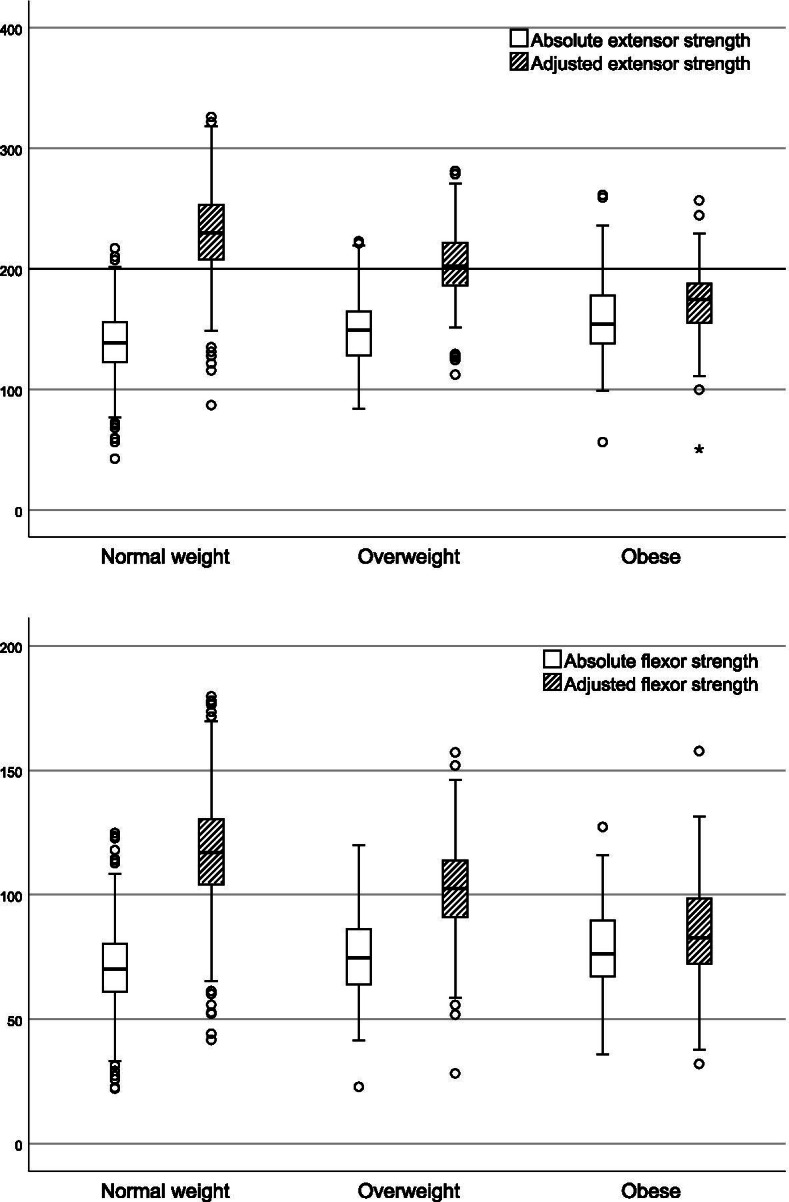
Knee extensor and flexor strength by FMI-group at baseline

To further understand the association between muscle strength and the body composition components, we firstly examined the relationship between thigh muscle strength and body fat mass, using the FMI classifications normal weight, overweight and obese. We found that absolute knee extensor and knee flexor strength was higher in overweight and obese women than in normal weight women (*p*<0.001). However, when adjusting for body weight the opposite was evident and muscle strength was significantly lower in those overweight and obese (*p*<0.001), (Table 2, Fig. 1).

**Table 2:** Muscle strength in relation to Fat Mass Index category at baseline, N=1036

	**Normal weight** (FMI ≤ 9 kg/m2)		**Overweight** (FMI >9-13 kg/m2)		**Obese** (FMI >13 kg/m^2^)		*P-*value*
	790	76%	187	18%	59	6%	
**Knee extensor (BL)**							
Absolute strength (Nm)	139.1 ^a^	(25.4)	148.4 ^b^	(26.8)	157.4 ^b^	(38.3)	<0.001
Weight-adjusted strength (%)	230.2 ^a^	(33.6)	203 ^a^	(28.9)	170.3 ^a^	(34.9)	<0.001
**Knee flexor (BL)**							
Absolute strength (Nm)	70.7 ^a^	(14.9)	75.1 ^b^	(16.3)	78.1 ^b^	(19.2)	<0.001
Weight-adjusted strength (%)	117 ^a^	(20.8)	102.8 ^a^	(19.5)	85.2 ^a^	(21.3)	<0.001

Reported values are mean (SD)

^*^P-value between groups, One-way ANOVA and Tukey post hoc test

^a^ Significantly different from both other groups;

^b^ Significantly different from the normal weight group

Thigh muscle strength, both extensors and flexors were highly correlated to percent lean mass in the legs (r_s_ = 0.57 and 0.50, *p*<0.001). Physical activity on the other hand, only weakly correlated with both muscle strength and mass (thigh muscle strength, r_s_ = 0.16 and 0.25; leg % lean mass, r_s_ =0.21, *p*<0.001 for all). Using linear regression analysis, we found that lean mass explained 34% of the variation in extensor strength and 26% in flexor strength, while adjustment for physical activity level had no or marginal effect (Table 3).

**Table 3:** Association between Leg lean mass and muscle strength at baseline, N=1036^a^

	**β**	**95% CI**	**R**^**2**^	***P***
A) Knee extensor strength				
Model 1. Leg Lean Mass (%)	813.7	743.9 - 883.5	0.34	<0.001
Model 2. Leg Lean Mass adjusted for physical activity level	801.2	729.6 - 872.9	0.34	<0.001
B) Knee flexor strength				
Model 1. Leg Lean Mass (%)	433.2	388.4 - 478	0.26	<0.001
Model 2. Leg Lean Mass adjusted for physical activity level	404.29	358.9 - 449.7	0.28	<0.001

^a^ Linear regression analysis

### Knee pain at age 35, muscle strength and body composition

**Fig. 2:**
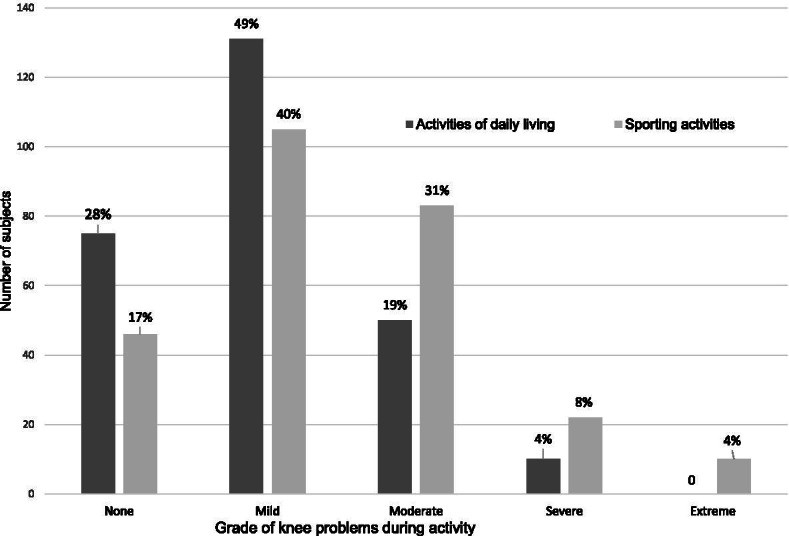
Perceived knee problems during activities in women with knee pain at follow-up. “At age 35 around one third of women reported knee problems during the previous month. As can be seen in the figure above, the majority of those were adversely affected. Activities of daily life were adversely affected in 72% and the ability to participate in their normal sporting activities was affected in 83%”

Knee complaints were common at age 35 with one third (33%) reporting pain, other knee symptoms and knee-related functional problems during the last month. Among them, 24% reported swelling, 52% grinding or clicking noises from the knee joint and 48% reported stiffness of various severity. Of those reporting knee complaints, the majority were adversely affected, 72% reported problems during everyday activities and 83% during sport and leisure activities (Fig. 2).

Comparing women with and without knee pain at follow-up, we found differences in thigh muscle strength and body composition. Women with knee pain tended to have lower muscle strength, at both knee extensors and flexors, but a slightly higher hamstrings-to-quadriceps strength ratio (mean (SD) 0.53 (0.11) and 0.51(0.08), *p*=0.04). The percentage of lean mass, both at the legs and total body was also lower. This is additionally reflected in a higher BMI and FMI. Sedentary lifestyle and physical activity were not associated with knee pain (*p*=0.18) and nor were knee injuries (*p*=0.19) or having a parent diagnosed with knee OA (38% vs 31%, *p* = 0.07) (Table 4). There was no correlation between muscle strength at age 25 and frequency of knee pain at age 35, (knee extensor r_s_ = -0.045, *p*= 0.20 and knee flexor r_s=_ -0.020, *p*= 0.58).

**Table 4:** Clinical characteristics of women WITH and WITHOUT knee pain at follow-up

		A) ALL women at follow-up *n*=797/728^a^		B) WITH Knee pain *n*=260		C) Without knee pain *n*=534		*P*-value^Ɨ^
		***Mean***	***(SD)***	***Mean***	***(SD)***	***Mean***	***(SD)***	
Muscle Strength (weight adjusted)								
Knee extensors (%)	714	192.8	(36.6)	184.9	(39)	196.8	(34.7)	<0.001
Knee flexors (%)	714	99.3	(22.4)	96.6	(23.1)	100.9	(21.7)	0.011
Hamstrings-to quadriceps ratio		0.52	(0.09)	0.53	(0.11)	0.51	(0.08)	0.044
Body Composition	n							
Weight (kg)	726	69.3	(13.2)	72.2	(15)	67.8	(12)	<0.001
BMI (kg/m^2^)	726	24.6	(4.5)	25.6	(5.2)	24.1	(4.0)	<0.001
FMI (Total body Fat Mass(kg)/height(m)^2^	726	8.5	(3.4)	9.2	(4)	8.2	(3)	0.004
Fat Mass-total body (%)	726	33.7	(0.07)	34.7	(8.1)	33.2	(6.6)	<0.001
Lean Mass-total body (%)	726	62.4	(0.07)	61.5	(7.8)	62.8	(6.4)	0.035
Lean Mass-Legs (%)	726	20.9	(0.02)	20.6	(2.2)	21.0	(1.9)	0.015
		***N***^***o.***^	***(%)***	***N***^***o.***^	***(%)***	***N***^***o.***^	***(%)***	
Physical activity level-High, n (%)	794	370	(46)	130	(50)	240	(45)	0.180
Physical activity level-Low, n (%)	794	424	(53)	130	(50)	294	(55)	
Knee injury, n (%)	797	120	(15)	45	(17)	74	(14)	0.193
Parent with knee OA n (%)	790	263	(33)	98	(38)	165	(31)	0.066

^a^ 728 participants attended for Biodex/DXA at follow-up; 797 returned questionnaires (knee health/physical activity); 794 answered questions about knee pain.

Comparison of women with and without knee pain used ^Ɨ^ Independent samples t-test for continuous variables and Chi-Square test for categorical variables

## Discussion

In this population-based study of young adult women, we explored the relationship between muscle strength, body composition and knee pain. Already among women in their mid-thirties, one third reported knee pain during the last month, a surprisingly high proportion for this age group, previous population studies show that knee complaints are highest in 55-74-year-old women [1, 32]. We found association between current muscle strength, lean mass and recent experience of knee pain at age 35, although lower strength at age 25 was not associated with future knee pain. In addition, we confirm a reciprocal relationship between BMI, the fat mass component of body composition and muscle strength, by which higher fat content and lower muscle strength are linked to knee pain already at this early age [33, 34]. Interestingly, the Chingford study, that followed British women over a 15 year period, found that higher BMI at baseline predicted later knee pain [33], although these women were in their mid-fifties at baseline.

As a risk factor for knee pain, we note that thigh muscle strength has already declined by around seven percent by the mid-thirties. This is normal according to Lindle et al [35] who found that concentric strength levels begin to decline in the fourth rather than the fifth decade, as was reported from other studies [36]. As the thigh muscles provide stability, proprioception and may reduce stress on the joint surface by attenuating impact loads [37], good muscle function may contribute to knee joint health. The hamstrings- to-quadriceps strength ratio has been suggested to be important for knee joint health, but we found no association between H:Q ratio and knee pain in this study.

A recent MRI study found that quadriceps and hamstrings strength was negatively associated with early changes in knee joint cartilage in young individuals at risk of knee OA [38]. Body composition influence muscle strength and we show that while women who were overweight or obese had higher absolute strength in their thigh muscles compared to those who were normal weight, adjustment for body weight inverted this relationship, as also indicated in previous studies [18]. Potentially this may be an early sign of sarcopenic obesity.

The percentage of total body fat mass was higher while percentage lean mass (total and regional) was lower at follow-up. It is well established that being overweight entails an increased risk for knee OA and lately, a body composition with greater percent fat mass and higher FMI has been suggested as a risk factor [20]. This comes not just from increased strain on the lower extremity joints, but through inflammatory activity induced by adipokines secreted from adipose tissue [39]. Interestingly, patients with radiographic knee OA have been shown to have a phenotype with higher bone mass, higher fat mass and lower lean body mass [19], of which the latter two were observed in women with knee pain in the current study.

In the study self-reported physical activity habits had changed so that considerably fewer regularly engaged in fitness activities, half of the women at age 35 compared with four out of five at 25. This more sedentary lifestyle could explain the altered body composition (less lean phenotype) [40] and lead to decreased lower extremity muscle strength and endurance [41]. Surprisingly, self-reported physical activity was the same in individuals with and without knee pain, which seems counter-intuitive since highly repetitive and high impact loading during sports has been associated with increased risk of knee OA, although meta-analyses are inconclusive [42]. Regular physical activity may have a direct positive effect on cartilage by stimulating joint nutrition [43], and supporting this, a positive association with tibial cartilage volume has also been seen in young adults [44]. Taken together, it suggests a complex relationship, likely influenced by longtime daily lifestyle and training habits.

In the current study muscle mass and strength strongly related, while physical activity did not appear to substantially contribute to either of these. While we cannot draw any conclusions about causality, this may reflect that few participants engaged in strength focused exercise, but rather on a wide range of activities involving aerobic fitness, flexibility or core stability. While strength in the thigh muscles may be maintained by moderate intensity physical activity for about thirty minutes most days a week [45], to increase lean mass this may be insufficient, as progressive resistance training during at least three months is required for muscle hypertrophy [23].

To the strengths of the study we attribute the longitudinal design, extensive investigations and the large number of women. The response rate is very good for this age group, still we cannot rule out the possibility of selection bias. However, comparing responders and non-responders at follow-up, only body weight and BMI differed (non-responders had higher weight and BMI), therefore we assume that the prevalence of knee pain might well be higher than we report. As we found no comparable studies on knee pain in healthy women at age 30-40, we believe that the current study is the first to show the high frequency of knee pain and the associations to body composition and muscle strength in this age-group. Limitations are acknowledged; firstly, knee pain was assessed only at follow-up. Secondly, knee pain and other symptoms were self-reported. Although subjective, the information provided is still valuable since the majority at this age are highly unlikely to seek medical advice for knee pain. While not part of the study protocol, a physical examination, would have given the opportunity to assess clinical knee OA-features such as joint malalignment, swelling and bony enlargement. A third limitation is that physical activity levels were self-assessed while an objective assessment with accelerometers would have given more detailed information. A second, 20-year follow-up, might answer if knee pain in the thirties is indicative of pre-stage knee OA and if body composition and thigh muscle strength at young age may predict knee OA. Finally, since the cohort consists of Caucasian women, the findings may not be applicable to other settings.

In conclusion, the novelty of this study is the finding that knee pain is common already among women in their mid-thirties, and affecting their daily activities. Lower thigh muscle strength in the mid-twenties was not associated with future knee pain, however women with knee pain tended to have lower thigh muscle strength and a body composition of higher body fat combined with lower lean mass. It suggests that maintaining a healthy body composition and adequate thigh muscle strength may be beneficial for knee joint health.

## Supplementary Information

**Additional file 1**. Knee problem questionnaire.

## Data Availability

The datasets used and/or analysed during the current study are available from the corresponding author on reasonable request.

## Abbreviations

OA: Osteoarthritis
BMI: Body Mass Index
FMI: Fat Mass Index
DXA: Dual Energy X-ray Absorptiometry
Nm: Newtonmeter
