# Supplementary material for: Knee pain in young adult women- associations with muscle strength, body composition and physical activity
Source: BMC Musculoskelet Disord. 2021 Aug 21;22:715. doi: 10.1186/s12891-021-04517-w (PMC8380389; doi:10.1186/s12891-021-04517-w)
Supplement: Supplementary file 1 — Additional file 1. Knee problem questionnaire. [file 12891_2021_4517_MOESM1_ESM.docx]

Additional file 1. Knee problem questionnaire, answered by 797 women at follow-up

|  | **Knee problem questionnaire** | **Response alternatives** |
| --- | --- | --- |
| 1 | How often do you experience knee symptoms? | Never, every month, every week, every day, always |
| 2 | How often do you experience knee pain? | Never, every month, every week, every day, always |
| 3 | Have your knee/knees been swollen during the last month? | Never, rarely, sometimes, often, always |
| 4 | Have you noticed a grinding or clicking noise from your knee during the last month? | Never, rarely, sometimes, often, always |
| 5 | What amount of stiffness have you experienced in your knee/knees in the morning during the last month? | None, mild, moderate, severe, extreme |
| 6 | What degree of knee problems have you experienced in every-day activities during the last month? | None, some, moderate, severe, extreme |
| 7 | What degree of knee problems have you experienced in sport-leisure activities during last month? | None, some, moderate, severe, extreme |
